# Long-term virological outcome in children receiving first-line antiretroviral therapy

**DOI:** 10.1186/s12981-018-0208-9

**Published:** 2018-11-26

**Authors:** Padmapriyadarsini Chandrasekaran, Anita Shet, Ramalingam Srinivasan, G. N. Sanjeeva, Sudha Subramanyan, Suba Sunderesan, Karunaianantham Ramesh, Bindu Gopalan, Elumalai Suresh, Navaneethan Poornagangadevi, Luke E. Hanna, Chockalingam Chandrasekar, Christine Wanke, Soumya Swaminathan

**Affiliations:** 1 Department of Clinic Research, ICMR-National Institute for Research in Tuberculosis, No. 1, Mayor Sathyamoorthy Road, Chetpet, Chennai, Tamil Nadu 600031 India; 2 Johns Hopkins Bloomberg School of Public Health, Baltimore, USA; 3 Indira Gandhi Institute of Child Health, Bangalore, India; 4 St Johns Research Institute, Bangalore, India; 5 Institute of Child Health and Government Hospital for Children, Chennai, India; 6 Government Hospital of Thoracic Medicine, Chennai, India; 7 Tufts University School of Medicine, Boston, USA; 8 Indian Council of Medical Research, New Delhi, India; 9 Present Address: World Health Organization, Geneva, Switzerland

**Keywords:** Antiretroviral therapy, Pediatrics, First-line, Treatment outcome, Viral load

## Abstract

**Background:**

Studies relating to long-term virological outcomes among children on first-line antiretroviral therapy (ART) from low and middle-income countries are limited.

**Methods:**

Perinatally HIV infected, ART-naive children, between 2 and 12 years of age, initiating NNRTI-based ART during 2010–2015, with at least 12 months of follow-up, were included in the analysis. CD4 cell counts and plasma HIV-1 RNA were measured every 24 weeks post-ART initiation. Immunologic failure was defined as a decrease in the CD4 count to pre-therapy levels or below and virologic failure as HIV-RNA of > 1000 copies/ml at 48 weeks after ART initiation. Genotypic resistance testing was performed for children with virologic failure. Logistic regression analysis was done to identify predictors of virologic failure.

**Results:**

Three hundred and ninety-three ART-naïve children living with HIV [mean (SD) age: 7.6 (3) years; mean (SD) CD4%: 16% (8); median (IQR) HIV-RNA: 5.1 (3.5–5.7) log_10_ copies/ml] were enrolled into the study. At 48 weeks, significant improvement occurred in weight-for-age and height-for-age z-scores from baseline (all p < 0.001). The immunologic response was good; almost 90% of children showing an increase in their absolute CD4^+^ T cell count to more than 350 cells/mm^3^. Immunological failure was noted among 11% (28/261) and virologic failure in 29% (94/328) of children. Of the 94 children with virologic failure at 12 months, 36 children showed immunologic failure while the rest had good immunologic improvement. There was no demonstrable correlation between virologic and immunologic failure. 62% had reported > 90% adherence to ART. At the time of virologic failure, multiple NNRTI-associated mutations were observed: 80%—K103N and Y181C being the major NNRTI mutations—observed. Sensitivity (95% CI) of immunologic failure to detect virologic failure was 7% (2–12), specificity 97% (92.4–98.9), PPV 44% (13.7–78.8) and NPV was 72% (65–77.9). There were no statistically significant predictors to detect children who will develop virologic failure on treatment.

**Conclusions:**

Considerable immunological improvement is seen in children with ART initiation, but may not be an effective tool to monitor treatment response in the long-term. There is a lack of correlation between immunologic and virologic response while on ART, which may lead to a delay in identifying treatment failures. Periodic viral load monitoring is, therefore, a priority.

## Background

Of the estimated 36.7 million people living with HIV worldwide in 2016, 6% (2.1 million) were children under 15 years of age [1]. An estimated 1.8 million individuals worldwide became newly infected with HIV in 2016 which includes 160,000 children under 15 years [2]. Children < 1 year of age are among the most vulnerable to HIV. As early initiation of antiretroviral therapy (ART) has shown benefits, the World Health Organization (WHO) has recommended initiation of ART for all children (< 10 years of age) and adolescents (10–19 years) living with HIV, regardless of WHO clinical stage or CD4 cell count [3]. Based on this recommendation, ART coverage in children living with HIV has increased in many countries resulting in a decrease in morbidity and mortality due to HIV infection [4].

Efficacy of ART is monitored by both clinical and laboratory measures, including estimation of HIV-1 viral load and CD4 cell count, while on treatment. WHO recommends viral load estimation as the preferred monitoring approach to diagnose and confirm treatment failure [5]. The 90-90-90 treatment targets call for 90% of those living with HIV to know their status, 90% of those who know their status to be on treatment, and 90% of those on treatment to be virally suppressed [6]. However, in low and middle-income countries, such monitoring has proved difficult given the inadequate laboratory facilities, shortage of trained staff and expensive reagents. The success of ART depends on the maintenance of long-term virological suppression, which is particularly challenging in children living with HIV. Varied response to different first-line regimens has been reported from pediatric observational cohorts from different regions of the world [7, 8, 9]. Despite the reduction in morbidity and mortality, a considerable proportion of patients fail to achieve a sustained virologic response to therapy [10, 11]. Thus treatment failure is an increasing concern.

Long-term data across all age groups of children are required to compare the effectiveness of ART regimens in resource-constrained settings. This will help us to identify the groups at high risk for virological failure while on ART and strategically plan interventions to overcome this hurdle and successfully manage children living with HIV. In India, till September 2016, approximately 56,000 children living with HIV were receiving free ART under the National Paediatric HIV/AIDS Initiative of the National AIDS Control Programme [12]. We evaluated the long-term outcome of children initiating first-line ART in India and assessed the factors associated with virological suppression or virological failure after 12-months of ART.

## Methods

Children initiating first-line ART in the “HIV-Associated Lipodystrophy Syndrome” (HALS) study, details are given elsewhere [13], and with at least 12 months of follow-up were included in this report. In brief, HALS study is a prospective study to determine the incidence of dyslipidemia and lipodystrophy among HIV-infected ART-naive children aged 2–12 years, initiating first-line ART as per the national guidelines and on follow-up during the period 2010–2016 at the National Institute for Research in Tuberculosis, Chennai, and St. John’s Hospital, Bangalore, India. The ART regimen consisted of two nucleoside reverse transcriptase inhibitors (NRTIs—lamivudine, and stavudine or zidovudine, based on the hemoglobin levels) and a non-nucleoside reverse transcription inhibitor (NNRTI, either nevirapine or efavirenz in children ≥ 3 years). Drug dosages were based on the body weight of the child and were given as tablets or syrups. Children were reviewed every 3 months after ART initiation.

### Data collection

During every review, the child’s well-being, adherence to ART and anthropometry were recorded. Adherence to ART was assessed by self-reporting by the caregiver, pill count and on-time hospital attendance by the child or his/her caregiver. Blood was drawn for complete blood count, CD4 percentage, absolute CD4^+^ T cell counts, and HIV-1 viral load, once every 6 months. CD4^+^ T cell percentage and CD4^+^ T cell counts were measured using the FACSCount flow cytometer (Beckton Dickinson Biosciences, San Jose, CA) and plasma viral load was estimated using the Roche COBAS AmpliPrep/Cobas TaqMan HIV-1 Test, v2.0. Stored plasma samples from children with at least 12 months of follow-up and proven virologic failure were analyzed for HIV drug resistance mutations (DRM) by HIV-1 RNA using the QIA amp viral RNA extraction kit (Qiagen, Valencia, CA) and sequencing the relevant portion of the HIV pol gene by adopting previously published HIV drug resistance testing protocol [14]. The sequences were assembled and analyzed using Seqscape software V2.6 (Applied Biosystems). HIV DRMs were defined using the Stanford University HIV drug resistance database.

### Outcome definition

Patient retention-rate was defined by the proportion of children who were still receiving care at the study sites. Attrition consisted mainly of mortality and loss to follow-up. If a child missed less than 4 doses of ART in a month, he/she was considered as > 95% adherent to ART. Similarly, if the child missed 5–12 doses of ART in a month they were considered only 80–95% adherent to ART, while missing more than 12 doses of ART in the last 1 month, put their adherence to < 80%. Loss to follow-up was defined as children not attending the scheduled follow-up visits for three consecutive visits, and all attempts to retrieve the patient for follow-up failed. The child also had not made any unscheduled visits during this period. The WHO Global Database on Child Growth and Malnutrition recommends a cut-off z-score of < − 2 standard deviation (SD) to classify low weight-for-age (WAZ) (underweight), low height-for-age (HAZ) (stunting), and low weight-for-height (WHZ) (wasting) as moderate, and a Z-score of < − 3 SD to define severe undernutrition [15]. Immunologic Failure (IF) was defined as either fall of CD4 count to pre-therapy baseline or below (2) > 30% fall of absolute CD4 count from the on-treatment peak value or (3) persistent CD4 levels below 100 cells/mm^3^; while immunologic recovery (IR) was defined as an increase in CD4^+^ T cell count of ≥ 25% from pre-therapy baseline levels [16, 17]. Virologic suppression was defined as plasma HIV-1 RNA < 400 copies/ml after 12 months of ART while virologic failure (VF) was defined as HIV-RNA over 1000 copies/ml after 12 months of ART in patients who had achieved initial virologic suppression [10, 18].

### Statistical analysis

The primary endpoints were virologic and immunologic failure at 12 months after initiation of first-line ART. The first analysis was done for all children who were on regular follow-up and completed 12 months of ART. Subsequently, a second analysis was done for those who went on to complete 24-months of ART. Data analysis was performed using SPSS software, version 19.0. We also calculated the changes in weight, height, and few laboratory parameters from baseline to weeks 48 and 96. We compared the continuous normally distributed data using Student t-test and proportions by Chi square test or McNemear test, as appropriate, to determine the change in anthropometry, immunology and virological parameters between pre-therapy baseline versus 12 months and pre-therapy baseline versus 24 months. We considered p < 0.0025 for statistical significance for comparing variables like stunting and underweight, moving from worst to better or normal category. This was done to avoid multiple comparisons for different time points. We performed a multivariate logistic regression analysis for all variables using enter method (all variables entered at the same time regardless of their significance level in univariate analysis), to identify independent predictors of virological failure at year 1. We considered age, gender, height and weight-for-age z scores, TB status, viral load, and CD4^+^ T cell count at the time of ART initiation, type of ART (nevirapine vs efavirenz), poor adherence (< 80% drug intake) to treatment, not having a parent as the primary caregiver, living in residential institutions as potential predictors of virological failure by entering all variable.

The study was approved by the Institutional Ethics Committee at both the institutions and informed written consent was obtained from the parent/guardian and assent from the child, wherever applicable.

## Results

During 2010–2015, 393 ART-naive HIV infected children were initiated on first-line ART at the study sites and included in the present analysis. The majority (97%) of them had acquired the infection perinatally; 49% were girls. Pre-exposure to single-dose nevirapine or other ART as part of prevention of mother to child transmission regimen could not be excluded for all children as some were either orphans staying at residential homes or with grandparents or distant relatives. Mean (SD) age of children at baseline was 7.6 (3) years, and median (IQR) HIV-1 RNA was 5.1 log_10_ copies/ml, (range: 3.5–5.7 log_10_ copies/ml). Undernutrition and poor immunological status were common among children before treatment initiation (Table 1).

**Table 1:** Baseline characteristics of 393 ART-naïve HIV infected children enrolled in the study

Characteristics of children	Values
Number of boys (%)	200 (51)
Mean age in years (SD)	7.6 (3.1)
Median WAZ score (IQR)	− 2.21 (− 2.7, − 1.5)
Median HAZ score (IQR)	− 2.1 (− 2.8, − 1.3)
Median WHZ score (IQR)	− 1.2 (− 1.9, − 0.5)
Median CD4 cell count (cells/mm^3^) (IQR)	388 (269–653)
Median HIV RNA viral load (copies/ml) (IQR)	141,000 (25,876–436,000)
Mean CD4% (SD)	16.55 (8.82)
WHO clinical stage	
1	42
2	98
3	150
4	65
CD4 cell count (cells/mm^3^)	n (%)
< 200	63 (16%)
200–349	102 (26%)
350–499	79 (20%)
> 500	147 (37%)
Viral load (copies/ml)	n (%)
< 400	10 (3%)
400–999	18 (5%)
> 1000	361 (92%)
Number of children on	
Efavirenz-based ART regimen	96 (24%)
Nevirapine-based ART regimen	289 (74%)
Other regimens (3NRTIs)	8 (2%)

*WAZ* weight for age, *HAZ* height for age, *WHZ* weight for height

### Patient in care cascade and adherence to ART

**Fig. 1:**
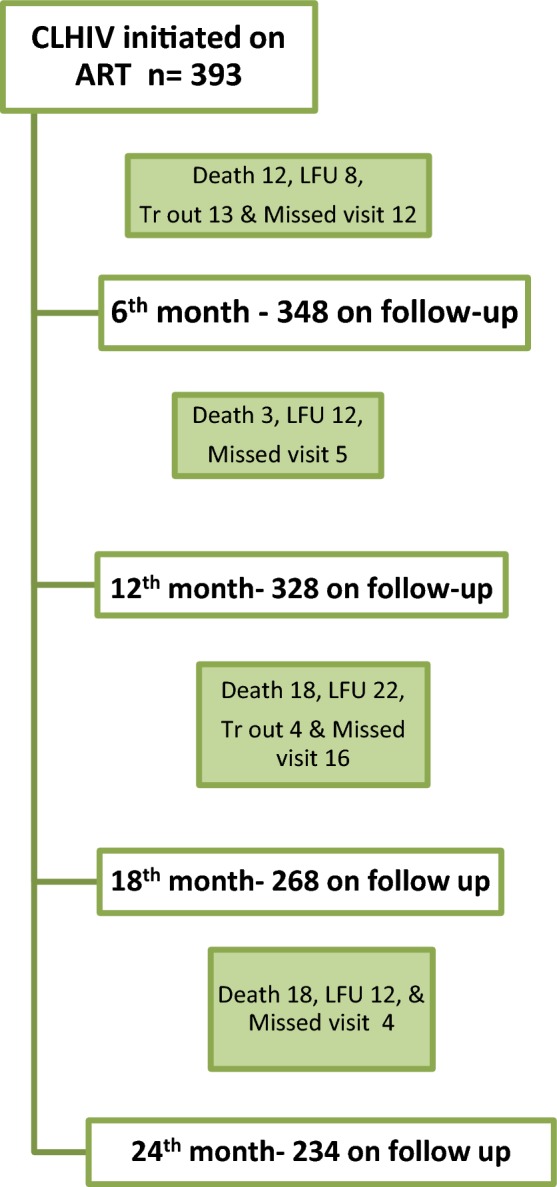
Schema showing the care cascade of children on ART in HALS study. *CLHIV* children living with HIV, *ART* antiretroviral therapy, *LFU* lost to follow-up, *Tr out* transfer out

Of the 393 children initiated on ART, 74% of children were started on nevirapine-based first-line ART while 24% on efavirenz-based first-line ART along with lamivudine and stavudine or zidovudine, (based on their hemoglobin levels > or < 6 gm/dl). The remaining 2% were on abacavir with lamivudine and stavudine or zidovudine. 378 children came for regular study-related follow-ups and further investigations. At the end of 12 months of ART, 328 of 393 children (83%) were still on follow-up with an overall retention rate of 87%. Self-reported adherence to ART at 12 months was > 90% drug intake in 83% (276/331) of children, 80–90% in 7% and < 80% in 10% of children on ART. There were—lost to follow-ups and missed visits during the follow-up period. By the end of 18 and 24 months of ART, there were 268 and 234 children on follow-up respectively. Figure 1 shows the study population along with reasons for the decrease in numbers during follow-up (care cascade).

### Clinical and immunologic follow-up

**Fig. 2:**
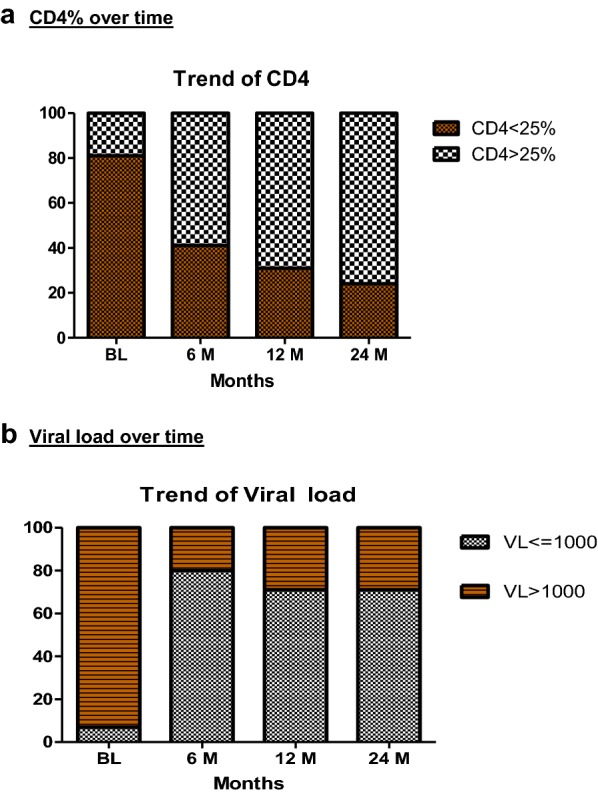
Stacked graph of the proportion of children on ART showing changes in **a** CD4% over time. **b** Viral load over time

After 12 months of ART, significant improvement was noticed in the weight-for-age and height-for-age z-scores from baseline (all p < 0.001). Though a significant number of children had moved from the worse stunting category to better and normal category, 21% of children remained stunted (HAZ score < − 2) and 22% undernourished for their age (WAZ score < − 2) (Table 2). The immunologic response was good; among children > 5 years of age, 90% [245/273] at 6 months, 91% [238/261] at 12 months, and 91% [176/193] at 24 months had an increase in their absolute CD4^+^ T cell count to more than 350 cells/mm^3^ when their baseline CD4 count was below 350 cells/mm^3^. A similar trend of increasing proportion of children showing a favorable response in CD4% with duration was seen as shown in Fig. 2. Immunologic failure was seen in 11% [28/261] and 9% [17/193] of children at 12 and 24 months respectively.

**Table 2:** Clinical and immunological outcome of children on ART during 24 months of follow-up

Laboratory parameters	Baseline (n = 393)	6 months (n = 340)	12 months (n = 323)	24 months (n = 234)
Mean (SD) hemoglobin (g/dl)	10.60 (1.64)	11.33 (1.46)	11.63 (1.44)	11.8 (1.3)
Mean (SD) CD4%	16.24 (7.93)	26.67 (9.54)	29.31 (9.47)	35.38 (36)^$^
Anthropometry n (%)	n = 393	n = 340	n = 323	n = 218
WAZ score < − 2	225 (57)	119 (35)	71 (22)*	16 (7)^$^
HAZ score < − 2	205 (52)	112 (33)	69 (21)*	17 (8)^$^
WHZ score < − 2	68 (17)	35 (10)	27 (8)*	23 (11)^$^
CD4%	n (%) = 345	n (%) = 284	n (%) = 243	n (%) = 135
< 25%	278 (81)	117 (41)	75 (31)*	33 (24)^$^
> 25%	67 (19)	170 (60)	168 (69)*	102 (76)^$^
CD4 cell count (> 5 years of age)	n (%) = 304	n = 273	n = 261	n = 193
> 350 cells/mm^3^	152 (50)	245 (90)	238 (91)*	176 (91)^$^
< 350 cells/mm^3^	152 (50)	28 (10)	28 (11)*	17 (9)^$^
Viral load (copies/ml)	n (%) = 389	n (%) = 348	n (%) = 328	n (%) = 218
< 400	11 (3)	273 (78)	221 (67)*	150 (69)^$^
400–1000	17 (4)	10 (3)	13 (4)	5 (2)
> 1000	361 (93)	70 (20)	94 (29)*	63 (29)^$^

*WAZ* weight for age, *HAZ* height for age, *WHZ* weight for height

* p < 0.05 between baseline and 12 months

^$^ p < 0.05 between baseline and 24 months by Mcnemar’s test

### Virological status at 12 and 24 months

Viral load data were available for 348 children at the end of 6 months, for 328 children at the end of 12 months and 218 children at the end of 24 months of ART. All the children (except 28) had a high viral load in the pre-ART period and showed a good clinical response after initiation of ART. 78% of children achieved undetectable viral load (< 400 copies/l as detected by our viral load machine) by 6 months of ART. This proportion decreased with increasing duration of ART—67% at 12 months and 69% at 24 months had undetectable plasma viral loads. (Table 2).

After 12 months of ART, a quarter of the children in this cohort (94/328) showed virological failure. Majority of failure (62/94) were seen in the age group of 7–12 years and in children receiving nevirapine-based ART (74/94, 79%) as compared to efavirenz-based ART (17/94, 18%) and other groups (3/94, 3%) (p = 0.192). The proportion of virologic failures remained similar at 24 months of ART too. 62% of children with VF reported > 90% adherence to ART.

### Drug resistance mutations

Of the 94 children with virological failure after 12 months of ART, drug resistance mutation (DRM) results were available for 65 children. At the time of virologic failure, 20% (13/65) of children had no DRM while 80% (52/65) of the children had multiple NNRTI-associated mutations.

**Fig. 3:**
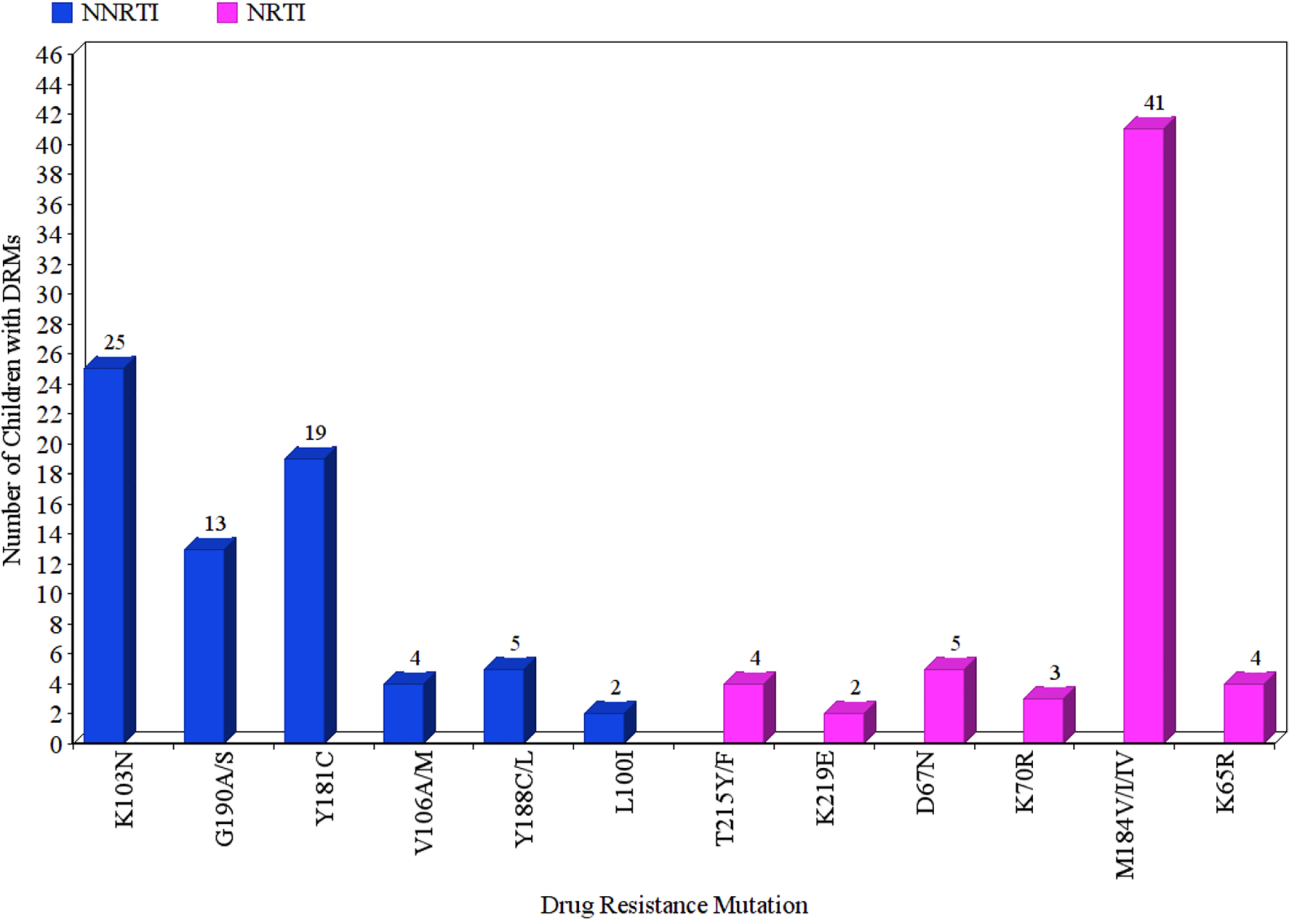
Major drug resistant mutations in HIV-1 isolates from the children at the time of virological failure

Among the children with DRMs, 85% (44/52) had both NRTI and NNRTI DRMs while 15% (8/52) had only major NNRTI DRMs. M184V/I was present in 79% (41/52) of the children, and 27% (14/52) had Thymidine analog mutations (TAMs). K103N (48%), Y181C (37%), G190A/S (25%), Y188C/L (10%), V106M/A (8%), K65R (8%) and L100I (4%) were the major NNRTI DRMs observed in these 52 children (Fig. 3). A retrospective DRM testing showed that only 6.4% (5/78) of the children had K103N as the major DRM at baseline (pre-ART). DRM results were available for 3 of these children at 12th month and they had either M184V or M184I mutation in addition to K103N at this time point.

### Virological–immunological discordance

Of the 94 children with virologic failure at 12 months, only 36 children showed immunologic failure. Correlation between virologic and immunologic failure was absent even at 24 months of ART (VF in 63 children versus IF in 39 children). Sensitivity (95% CI) of immunologic failure to detect virologic failure was only 7% (2.0–12.0), specificity 97% (92.4–98.9), PPV 44% (13.7–78.8) and NPV was 72% (65.0–77.9), highlighting the fact that immunologic failure cannot detect virologic failure accurately and hence cannot be a good tool to assess the treatment response in children on ART.

At the time of virologic failure, none of the children were switched to second-line ART but continued on first line regimen as their CD4^+^ T cell counts were high and they did not fit into the criteria for switching to second-line ART as per the national guidelines [19].

### Predictors of virologic failure

In univariate analysis, the type of ART regimen showed a trend towards positive correlation with virologic failure. But in multivariate analysis, there were no identifiable predictors to detect children who will develop virologic failure while on treatment (Table 3).

**Table 3:** Factors associated with virologic failure in children on antiretroviral therapy

Variable	Univariate analysis		Multivariate analysis	
	Odds ratio (95% CI)	p value	Odds ratio (95% CI)	p value
Age (years)				
≤ 5		0.63		0.97
> 5	1.19 (0.50–1.74)		1.02 (0.45–2.26)	
Gender				
Girls		0.81		0.62
Boys	1.07 (0.57–2.01)		0.85 (0.45–1.59)	
ART regimen				
Nevirapine		0.06		0.48
Efavirenz	2.15 (0.88–5.38)		1.77 (0.36–8.67)	
Primary caregiver				
Yes		0.06		0.26
No	0.42 (0.15–1.13)		0.58 (0.21–1.52)	
Staying in a residential institution				
Yes		0.71		0.46
No	1.15 (0.49–2.68)		1.36 (0.58–3.19)	
TB status				
No		0.09		0.95
Yes	0.50 (0.20–1.21)		0.95 (0.19–4.73)	
CD4 at baseline				
< 350 cells/mm^3^		0.16		0.18
> 350	1.53 (0.80–2.91)		1.56 (0.80–3.00)	
Viral load at baseline (copies/ml)				
≤ 400		0.51		0.24
> 400	2.48 (0.0–92.47)		3.49 (0.42–28.8)	
WAZ score				
> − 2.00		0.23		0.25
≤ − 2.00	0.69 (0.36–1.38)		0.62 (0.28–1.39)	
HAZ score				
> − 2.00		0.51		0.78
≤ − 2.00	0.82 (0.43–1.55)		0.89 (0.42–1.91)	

*WAZ* weight for age z-score, *HAZ* height-for-age z-score

## Discussion

In summary, the study revealed improvement in clinical, immunological and virological outcomes among HIV infected children after 96-weeks of NNRTI-based ART. However, a quarter of them had developed virologic failure by the end of 1-year of ART, which did not reflect in their immunological parameters, i.e., CD4 cell counts. Monitoring of individuals on ART is important to ensure treatment efficacy and improved health outcomes. Hence periodic monitoring of viral load at least once in 6 months after initiation of NNRTI-based ART, will allow early identification of virologic failures and regimen change, thus avoiding accumulation of resistant strains and treatment failures. WHO recommends routine viral load testing at 6 and 12 months after ART initiation and every 12 months thereafter as the preferred monitoring timelines to diagnose and confirm treatment failure [20].

After 12–24 months of NNRTI-based ART, significant improvement was observed in the height and weight-z-scores, CD4% and CD4 cell counts of HIV-infected Indian children, similar to that observed in the Thai and Kenyan cohorts [21, 22]. However, in our cohort, a substantial number of children remained stunted even after 1-year of ART. The reasons for this could be either their perinatal exposure to HIV and subsequent suppression of growth potential or continued to be immunosuppressed or the late initiation of ART with an advanced stage of the disease [23, 24].

In our cohort, 90% of children above 5 years and about 70% of younger children (< 5 years) showed immune recovery by 48 weeks. Thai cohort also noticed such high rates of immune recovery [21]. The lower rates of immune recovery in the younger age group could be due to under-dosing of ART as proper child-friendly formulations of ART were not available in the programme then. At the end of 48 weeks, 67% of children in our cohort had undetectable plasma HIV-1 RNA. This was slightly lower than previous reports of 70–75% [21, 25]. Non-response to treatment due to initiation of ART at an older age secondary to a delayed diagnosis of the disease or advanced stage of the disease as evidenced by low CD4 cell counts could attribute to a lesser proportion of children showing virological suppression. An additional 4% showed a viral load between 400 and 1000 copies/ml at the 48th week; who showed virological suppression at a subsequent visit, probably delayed converters.

Literature reports varying rates of VF; 20–33% in Thai paediatric-HIV cohorts [18, 21], 38% in a South African cohort [10], and 33% among children in Mali > 1-month post-NNRTI-based ART initiation [26]. In our cohort, at the end of 48 weeks of ART, 29% of children (94 children) experienced VF. Majority of the VF occurred between 6 and 12 months of ART and most of them occurred in children on Nevirapine-based ART, raising the issue of sub-therapeutic concentration of nevirapine during the lead-in period of treatment [27]. Failure to detect VF early and continuation on the failing regimen may result in accumulation of resistant viruses leading to clinical deterioration and death. Hence monitoring of viral load, at least once in 6 months after initiation of NNRTI-based ART, will allow early identification of VF and regimen change if warranted. Also, viral load monitoring can be instrumental in identifying those in need of additional adherence support and preserving treatment options.

Unlike other studies, our cohort had a low rate (6%) of pre-treatment NNRTI resistance mutation. Crowell et al. reported 38% baseline NNRTI resistance among 120 treatment-naive HIV-infected children in Mali, while Kityo et al. reported 17% pre-treatment drug resistance among 278 children with HIV [26, 28]. Though mother-to-child transmission was the commonest mode of transmission of HIV infection to the child, the low rate of pre-treatment NNRTI mutation could be due to the poor uptake of single-dose nevirapine by pregnant mothers as the majority of them had deliveries at home and approached the health care system only after delivery. Among those who developed VF, M184V, K103N, and Y181C were the most common DRMs observed as in South Africa [29].

Of the 94 children with VF, only ten showed concurrent IF. The sensitivity of IF to detect treatment failure was low in our study, similar to other studies where it varied between 3 and 4% [21, 30]. Gunda et al. [17] also reported low sensitivity and PPV of WHO immunological criteria to detect treatment failure. There is a time lag of approximately 6–12 months between the onset of VF and IF and subsequent clinical failure. The poor sensitivity of IF in detecting treatment failure may cause physicians to miss early detection of treatment failure and drug resistance. Unfortunately, in many low- and middle-income settings, routine HIV-1-RNA monitoring is still unavailable and they rely on CD4 counts for monitoring treatment response. Countries have to make available periodic viral load testing for children living with HIV if they have to achieve 90-90-90 target of UNAIDS. Also, if expertise for pediatric venipuncture is not available, the techniques to use dried blood spot specimens, cheaper in-house techniques to estimate viral loads should be made available [31].

Various predictors of VF have been identified and reported in the literature, which include younger age [30], low CD4% at the time of ART initiation [30], type of ART [26, 30], baseline NNRTI resistance [26, 28], etc. Father as the main caregiver of the child and lack of access to tap water; have also been identified as risk factors for VF [32]. Njom et al. [33] reported that being motherless children (motherless orphanhood) was associated with VF (aOR: 2.9, 95% CI 1.3–6.1). However, in our cohort, we were not able to identify any specific predictors for virologic failures, though we noticed a trend among children with low CD4 cell counts at the time of ART initiation, for developing virological failure (OR = 0.999, 95% CI 0.98–1.00, p = 0.02).

Emotional and developmental issues particular to elder children may also pose difficulties in the daily administration of ART, affecting adherence and virological suppression. Unfortunately, the ART adherence recorded by self-reporting during the monthly visits of children to the clinic, fail to capture these missed doses. Hence, adherence to ART should be emphasized during every clinic visit to both the children as well as their caregivers.

The strength of this study is the long-term virological outcome data of children living with HIV initiated on first-line ART in a resource-constrained setting. Limitation of this cohort was that a repeat viral load testing after 1 month could not be done in all children with a viral load of 400–1000 copies while on ART. Also, not all children followed up to 24 months post ART initiation had their viral load measurements done either because they were lost to follow-up or due to a shortage of viral load kits. Our findings highlight the need for the healthcare system to strengthen its infrastructure and supply management if routine viral load monitoring services are to be made available in the national pediatric ART programmes.

## Conclusions

CD4 criteria to detect treatment failures perform poorly as they fail to detect virological failures early—on during treatment. Using CD4 criteria to monitor treatment response to ART in low and middle-income countries may result in misclassification, and delay in treatment switches. Plasma HIV-1 RNA detection along with adherence counseling should be done periodically as a priority, to ensure proper monitoring of children receiving ART. This will not only improve treatment outcome but also reduce the development of drug resistance in children on ART.

## Abbreviations

ART: antiretroviral therapy
DRM: drug-resistant mutations
EFV: efavirenz
HALS: HIV associated lipodystrophy syndrome
HAZ: height-for-age z score
HIV: human immunodeficiency virus
IF: immunologic failure
IQR: interquartile range
NNRTI: non-nucleoside reverse transcription inhibitor
NPV: negative predictive value
NRTI: nucleoside reverse transcription inhibitor
NVP: nevirapine
PPV: positive predictive value
RNA: ribonucleic acid
SD: standard deviation
TAM: thymidine analog mutations
VF: virologic failure
UNAIDS: United Nations Programme on HIV/AIDS
WAZ: weight-for-age Z-score
WHZ: weight-for-height Z-score

## References

[1] Global and Regional Trends—UNICEF DATA: monitoring the situation of children and women. https://data.unicef.org/topic/hivaids/global-regional-trends/. Accessed 23 June 2018.

[2] The Global HIV/AIDS Epidemic. https://www.hiv.gov/federal-response/pepfar-global-aids/global-hiv-aids-overview. Accessed 20 Oct 2018.

[3] World Health Organization. WHO early release guidelines on when to start antiretroviral therapy and on pre-exposure prophylaxis for HIV September 2015. Geneva: World Health Organization; 2015. http://www.who.int/hiv/pub/guidelines/en/. Accessed 28 June 2018. 26598776

[4] Koye DN, Ayele TA, Zeleke BM (2012). Predictors of mortality among children on antiretroviral therapy at a referral hospital, Northwest Ethiopia: a retrospective follow up study. BMC Pediatr.

[5] Hammer SM, Saag MS, Schechter M, Montaner JS, Schooley RT, Jacobsen DM (2006). Treatment for adult HIV infection: 2006 recommendations of the international AIDS society-USA panel. JAMA.

[6] UNAIDS 90-90-90: treatment for all. http://www.unaids.org/en/resources/909090. Accessed 23 June 2018.

[7] Sebunya R, Mussime V, Kitaka SB, Ndeezi G (2013). Incidence and risk factors for first-line antiretroviral treatment failure among Ugandan children attending an urban HIV clinic. AIDS Res Ther.

[8] Lowenthal ED, Ellenberg JH, Machine E, Sagdeo A, Boiditsew S, Steenhoff AP (2013). Association between African based compared with nevirapine-based antiretroviral regimens and virological failure in HIV infected children. JAMA.

[9] Kukoyi O, Renner L, Powell J, Barry O, Prin M, Kusah J (2016). Viral load monitoring and antiretroviral treatment outcomes in a pediatric HIV cohort in Ghana. BMC Infect Dis.

[10] Barth RE, Tempelman HA, Smelt E, Wensing AM, Hoepelman AI, Geelen SP (2011). Long-term outcome of children receiving antiretroviral treatment in rural South Africa. Pediatr Infect Dis J.

[11] Duong T, Judd A, Collins IJ, Doerholt K, Lyall H, Foster C (2014). Long-term virological outcome in children on antiretroviral therapy in the UK and Ireland. AIDS.

[12] National AIDS Control Organisation. National paediatric initiative on HIV-AIDS. http://www.naco.gov.in/pressrelease/national-paediatric-initiative-hiv-aids-launched. Accessed 28 June 2018.

[13] Padmapriyadarsini C, Shet A, Srinivasan R, Ramachandran G, Sanjeeva GN, Devi P (2018). High prevalence of lipid abnormalities and insulin resistance among antiretroviral naïve HIV-infected children in India. Pediatr Infect Dis J.

[14] WHO manual for HIV drug resistance testing using dried blood spot specimens, July 2012. http://www.who.int/hiv/pub/drugresistance/dried_blood_spots/en/. Accessed 5 July 2016.

[15] WHO Global Database on Child Growth and Malnutrition. Compiled by Mercedes de Onis and Monika Blössner Programme of Nutrition. Geneva:World Health Organisation; 1997. http://apps.who.int/iris/bitstream/handle/10665/63750/WHO_NUT_97.4.pdf;jsessionid=E8549D8861C7DC1EA9079BF6B8F98AD5?sequence=1. Accessed 28 June 2018.

[16] WHO. Consolidated guidelines on the use of antiretroviral drugs for treating and preventing HIV infection: recommendations for a public health approach. Geneva: World Health Organization; 2013. https://www.ncbi.nlm.nih.gov/pubmed/24716260. Accessed 30 June 2018. 24716260

[17] Gunda DW, Kidenya BR, Mshana SE, Kilonzo SB, Mpondo BCT (2017). Accuracy of WHO immunological criteria in identifying virological failure among HIV-infected adults on first-line antiretroviral therapy in Mwanza, North-western Tanzania. BMC Res Notes.

[18] Jittamala P, Puthanakit T, Chaiinseeard S, Sirisanthana V (2009). Predictors of virologic failure and genotypic resistance mutation patterns in Thai children receiving non-nucleoside reverse transcriptase inhibitor-based antiretroviral therapy. Pediatr Infect Dis J.

[19] National Guidelines on Second-line and Alternative First-line ART for Adults and Adolescents. May 2013. National AIDS Control Organization. Ministry of Health and Family Welfare. Government of India. http://www.naco.gov.in/sites/default/files/National%20Guidelines%20on%20Secondline%20and%20Alternative%20Firstline%20ART%20For%20Adults%20and%20Adolescents%20May%202013_0.pdf. Accessed 1 July 2018.

[20] WHO. What’s new in treatment monitoring: viral load and CD4 testing—HIV treatment and care. July 2017. http://apps.who.int/iris/bitstream/handle/10665/255891/WHO-HIV-2017.22-ng.pdf?sequence=1. Accessed 20 Oct 2018.

[21] Bunupuradah T, Puthanakit T, Kosalaraksa P, Kerr S, Boonrak P, Prasitsuebsai W (2011). Immunologic and virologic failure after first-line NNRTI-based antiretroviral therapy in Thai HIV infected children. AIDS Res Ther.

[22] Wamalwa DC, Farquhar C, Obimbo EM, Selig S, Mbori-Ngacha DA, Richardson BA (2007). Early response to highly active antiretroviral therapy in HIV-1-infected Kenyan children. J Acquir Immune Defic Syndr.

[23] Joel DR, Mabikwa V, Makhanda J, Tolle MA, Anabwani GM, Ahmed SF (2014). The prevalence and determinants of short stature in HIV-infected children. J Int Assoc Provid AIDS Care.

[24] Arpadi S, Horlick M, Shane E (2004). Metabolic bone disease in human immunodeficiency virus-infected children. J Clin Endocrinol Metab.

[25] Ciaranello AL, Chang Y, Margulis AV, Bernstein A, Bassett IV, Losina E (2009). Effectiveness of pediatric antiretroviral therapy in resource-limited settings: a systematic review and meta-analysis. Clin Infect Dis.

[26] Crowell CS, Maiga AI, Sylla M, Taiwo B, Kone N, Oron AP (2017). High rates of baseline drug resistance and virologic failure among ART-naïve HIV infected children in Mali. Pediatr Infect Dis J.

[27] Gopalan BP, Mehta K, D’souza RR, Rajnala N, HK AK, Ramachandran G (2017). Sub-therapeutic nevirapine concentration during antiretroviral treatment initiation among children living with HIV: implications for therapeutic drug monitoring. PLoS ONE.

[28] Kityo C, Boerma RS, Sigaloff KCE, Kaudha E, Calis JCJ, Musiime V (2017). Pretreatment HIV drug resistance results in virological failure and accumulation of additional resistance mutations in Ugandan children. J Antimicrob Chemother.

[29] Pillay S, Bland RM, Lessells RJ, Manasa J, de Oliveira T, Danaviah S (2014). Drug resistance in children at virological failure in a rural KwaZulu-Natal, South Africa, cohort. AIDS Res Ther.

[30] Emmett SD, Cunningham CK, Mmbaga BT, Kinabo GD, Schimana W, Swai ME (2010). Predicting virologic failure among HIV-1-infected children receiving antiretroviral therapy in Tanzania: a cross-sectional study. J Acquir Immune Defic Syndr.

[31] Neogi U, Gupta S, Rodrigues R, Sahoo PN, Rao SD, Rewari BB (2012). Dried blood spot HIV-1 RNA quantification: a useful tool for viral load monitoring among HIV-infected individuals in India. Indian J Med Res.

[32] Amani-Bosse C, Dahourou DL, Malateste K, Amorissani-Folquet M, Coulibaly M, Cattes S (2017). Virological response and resistances over 12 months among HIV-infected children less than two years receiving first-line lopinavir/ritonavir-based antiretroviral therapy in Cote d’Ivoire and Burkina Faso: the MONOD ANRS 12206 cohort. J Int AIDS Soc.

[33] Njom Nlend AE, Motaze AN, Ndiang ST, Fokam J (2017). Predictors of virologic failure on first-line antiretroviral therapy among children in a referral pediatric center in Cameroon. Pediatr Infect Dis J.

